# Lipid Metabolism and Ferroptosis Resistance in Dormant Breast Cancer Cells: Emerging Therapeutic Vulnerabilities

**DOI:** 10.3390/diagnostics16050667

**Published:** 2026-02-25

**Authors:** Giulia Capella, Fulvio Borella, Eleonora Battista, Niccolò Gallio, Mathilde Hotot, Luca Bertero, Paola Cassoni, Isabella Castellano

**Affiliations:** 1 Pathology Unit, Department of Medical Sciences, University of Turin, 10126 Turin, Italy; eleonora.battista@unito.it (E.B.); mathilde.hotot@unito.it (M.H.); isabella.castellano@unito.it (I.C.); 2 Gynecology and Obstetrics 1U, Departments of Surgical Sciences, University of Turin, 10126 Turin, Italy; 3 Gynecology and Obstetrics 2U, Departments of Surgical Sciences, University of Turin, 10126 Turin, Italy

**Keywords:** breast cancer, dormancy, ferroptosis, lipid metabolism, ACSL3, SCD1, lipid droplets, GPX4, glutathione, MUFAs, therapeutic targets

## Abstract

Late metastatic relapses still represent a major clinical challenge in breast cancer, particularly in hormone receptor-positive (HR+) disease, with dormant disseminated tumor cells (DTCs) playing a critical role in driving late metastatic relapses. In fact, these cells can persist in a quiescent, non-proliferative state in metabolically hostile microenvironments such as the bone marrow, where they can resist conventional therapies, driving metastatic relapses even years after primary tumor removal. Recent advances highlight the crucial role of lipid metabolism in protecting dormant DTCs from ferroptosis—a form of regulated cell death characterized by iron-dependent lipid peroxidation. Dormant DTCs can avoid lipid peroxidation by incorporating monounsaturated fatty acids (MUFAs) into membrane phospholipids through ACSL3 and SCD1 activity, while accumulating lipid droplets (LDs) that sequester oxidizable polyunsaturated fatty acids (PUFAs), thus limiting the substrates available for ferroptosis. In parallel, antioxidant systems such as the GPX4–glutathione axis further prevent lethal lipid-derived reactive oxidative species (ROS) accumulation. This review highlights the central role of lipid metabolism, redox regulation and ferroptosis resistance in dormant DTCs; it also explores emerging therapeutic opportunities to overcome dormancy-associated resistance and reduce late relapse risk in breast cancer.

## 1. Introduction

Breast cancer (BC) is one of the most prevalent malignancies worldwide and remains the leading cause of cancer-related mortality among women aged 20 to 50 years [1,2]. Approximately 75% of all BC cases are characterized by the expression of the estrogen receptor (ER). This crucial nuclear marker, together with the progesterone receptor (PR) and the cell surface receptor human epidermal growth factor receptor 2 (HER2), represent the basis of current diagnosis, prognostic assessment and therapeutic stratification. The identification of all these biomarkers guides the clinical management of BC patients by aiding the stratification into molecular subtypes and enabling a truly tailored treatment approach, in particular endocrine therapy (for ER/PR-positive tumors) and targeted anti-HER2 regimens (for HER2-positive tumors) [3,4]. Despite significant advances in personalized therapeutic strategies—including surgery, radiation, chemotherapy, and molecularly targeted approaches—the disease recurrence and the acquisition of therapeutic resistance still represent major clinical challenges. For these reasons, understanding the underlying mechanisms that drive tumor relapse is critical both for improving long-term patient outcomes and their clinical management [3,4]. In particular, in patients who experience metastatic relapse years or even decades after apparent clinical remission, the presence of tumor cells that can escape the first line of treatment seems to be related to late relapses. Specifically, dormant disseminated tumor cells (DTCs) have recently gained interest and have been described as key contributors to late relapses, since they can enter a state of dormancy, remaining clinically undetectable for years [5]. These peculiar cells persist in a dormant, non-proliferative state within niches in secondary organs, and are often resistant to conventional therapies. In fact, different studies highlight their ability to survive in these distant tissues through the acquisition of unique survival programs, which enable DTCs to survive in a stressful environment, making them a critical obstacle to disease eradication [6,7]. Therefore, the long latency period associated with tumor dormancy requires specific strategies that can target these cells before they exit quiescence and start metastatic outgrowth [5,6,7].

Among breast cancer subtypes, dormancy is of particular interest in hormone receptor-positive (HR+) lesions, which are characterized by a prolonged risk of recurrence, that can occur many years after initial treatment [8,9]. In HR+ tumors, DTCs may reside in a quiescent state within bone marrow or other metastatic niches, where they are able to evade immune surveillance and resist endocrine therapy [10]. The specific crosstalk between DTCs and the cellular components of these niches—including endothelial, stromal and immune cells—has been shown to supply essential survival signals and provides protection against drug-induced toxicity. For example, DTCs occupying perivascular niches interact with endothelial and extracellular matrix components that shield them from chemotherapy effects. Also, signals from stromal and immune-suppressive cells within bone marrow and other metastatic microenvironments, support both dormancy and resistance to cytotoxic therapies [11,12].

In order to survive in such metabolically challenging microenvironments, these cells rely on diverse, extensive metabolic adaptations that support their long-term survival. While signaling pathways regulating cell-cycle arrest, immune evasion, and niche interactions during dormancy have been extensively characterized, the metabolic adaptation that enable DTCs to survive in hostile niches are still poorly understood. Emerging evidence highlights metabolic plasticity—the ability to rewire metabolic networks in response to environmental stress—as a key feature of DTCs, and points to lipid metabolic rewiring as a crucial adaptive strategy supporting survival under nutrient deprivation, oxidative stress and therapy-induced challenges [13,14,15].

Lipids can be used not only as energy substrates through β-oxidation, but they also play essential structural and signaling roles that support cell membrane integrity, redox homeostasis, and long-term quiescence. In particular, structural lipids such as glycerophospholipids and sphingolipids are key constituents of biological membranes, maintaining fluidity and stability, while lipid-derived signaling molecules modulate pathways involved in cell survival and stress responses. Moreover, lipid metabolism contributes to cellular redox balance and adaptive responses to environmental stress in cancer cells [16,17,18].

Specifically, dormant breast cancer cells have been shown to shift their metabolism toward enhanced fatty acid oxidation (FAO), lipid droplet accumulation, and suppression of de novo lipogenesis, thereby enabling adaptation of their lipid profile to resist oxidative stress and nutrient deprivation. These mechanisms enable DTCs to preserve viability under challenging conditions [19,20]. This reliance on FAO provides a steady energy supply, while minimizing the generation of reactive oxygen species (ROS) associated with high glycolytic flux. Furthermore, the stored lipids within droplets protect the cells from lipotoxicity by sequestering potentially toxic fatty acids [21]. This metabolic shift is essential for keeping the low proliferation rate, which is a typical feature of the dormant state, while at the same time the metabolism is still active, thus contributing to maintaining tolerance against stress.

In this specific context, even if de novo lipogenesis associated with proliferative programs is generally suppressed during dormancy, dormant DTCs selectively retain or activate specific lipid metabolic pathways that sustain membrane remodeling, lipid storage, and redox homeostasis, thus supporting long-term survival. This apparent discrepancy likely reflects context-dependent regulation, influenced by metastatic niches (e.g., bone marrow), disease stage, and breast cancer subtype.

An important consequence of lipid metabolic rewiring is its impact on ferroptosis, which is a regulated, iron-dependent form of cell death driven by lipid peroxidation. This biological process is biochemically defined by the iron-catalyzed accumulation of lethal lipid peroxides within cellular membranes [22,23]. In fact, unlike apoptosis or necroptosis, this mechanism is closely related to the availability and oxidation status of specific cell membrane lipids, which are the polyunsaturated fatty acids (PUFAs), making it a unique process of interest in cancer biology and therapy. Consequently, lipid remodeling and redox balance emerge as key determinants of sensitivity to this mechanism [24,25,26]. Interestingly, DTCs appear to adopt strategies to avoid ferroptosis, such as upregulating antioxidant defenses (e.g., GPX4 activity), remodeling their lipid content to reduce peroxidation-prone species, such as PUFAs, and reinforcing lipid storage mechanisms [25,26].

Understanding how dormant DTCs escape ferroptosis through lipidic metabolic rewiring could unveil actionable vulnerabilities. In fact, targeting these metabolic adaptations can offer promising novel therapeutic approaches to selectively eliminate dormant cells before they exit quiescence, thus starting the metastatic outgrowth. However, their rarity and technical inaccessibility represent an obstacle to direct functional studies on dormant DTCs isolated from patients, and for these reasons many mechanistic insights currently rely on preclinical models.

This review aims to comprehensively summarize the current understanding of how metabolic rewiring, with a particular focus on lipid metabolism, is involved in ferroptosis resistance in dormant DTCs, and to discuss emerging therapeutic strategies that could exploit these vulnerabilities to reduce the risk of late metastatic recurrence.

## 2. Lipid Metabolic Rewiring Protects Dormant Breast Cancer Cells from Ferroptosis via ACSL3-Mediated MUFAs Incorporation

**Figure 1:**
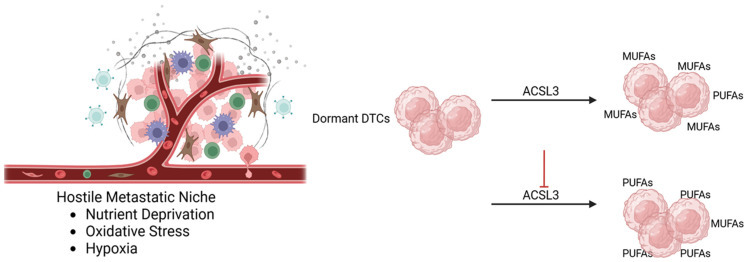
Membrane lipid remodeling in dormant disseminated tumor cells (DTCs). Dormant disseminated tumor cells (DTCs) remodel membrane lipid composition through acyl-CoA synthetase long-chain family member 3 (ACSL3)-mediated fatty acid activation, and incorporation of exogenous monounsaturated fatty acids (MUFAs) into membrane phospholipids. This process limits the availability of oxidable polyunsaturated fatty acids (PUFAs) and reduces susceptibility to lipid peroxidation and ferroptosis. Inhibition of ACSL3 decreases MUFAs enrichment and sensitizes DTCs to ferroptosis. Created in BioRender. Bertero, L. (2026) https://BioRender.com/fengpv4.

Dormant DTCs often undergo important lipid metabolic rewiring to survive under condition of nutrient deprivation and oxidative stress within metastatic niches. A pivotal adaptative mechanism involves the reshaping of membrane lipid composition, in order to reduce damages from lipid peroxidation, which is a key trigger of ferroptosis. Specifically, these cells selectively incorporate monounsaturated fatty acids (MUFAs), such as oleic acid, into membrane phospholipids, which are intrinsically less susceptible to lipid peroxidation compared to polyunsaturated fatty acids (PUFAs) [14,27]. This enrichment in MUFAs is associated with a relative reduction in PUFAs abundance; limiting the availability of peroxidation-prone lipid substrates can effectively limit ferroptosis induction (Figure 1).

A key enzyme involved in this protective remodeling is acyl-CoA synthetase long-chain family member 3 (ACSL3), which preferentially activates exogenous MUFAs by converting them into fatty acyl-CoA intermediates. However, increased lipid synthesis in dormant DTCs should not be interpreted as a reactivation of proliferative de novo lipogenesis, but rather as a functional rewiring promoting ferroptosis resistance.

These activated fatty acids are essential for the biosynthesis and remodeling of membrane phospholipids, helping to shape a lipid composition less vulnerable to peroxidation and consequently to ferroptosis death [27,28]. So, in this context, ACSL3 plays an opposing role when compared to ACSL4, which instead promotes PUFAs incorporation into phospholipids and enhances ferroptosis sensitivity. Interestingly, elevated ACSL3 expression has been consistently observed in preclinical models of HR+ breast cancer dormancy, where it strongly correlates with reduced lipid peroxidation and increased ferroptosis resistance [28].

In addition, this lipid rewiring is not just related to the tumor cells, but it is also supported by a metabolic crosstalk with the tumor microenvironment. Specifically, metastatic niches that are enriched in adipocytes, such as the mammary fat pad or bone marrow, provide abundant exogenous MUFAs—especially oleic acid—that dormant DTCs efficiently uptake and incorporate into their membranes through ACSL3-mediated activation and phospholipids remodeling. Consequently, this exogenous fatty acid supply further enhanced protection against oxidative stress and ferroptosis [29]. This metabolic crosstalk between stromal adipocytes and dormant DTCs highlights the critical role of the microenvironment in both sustaining dormancy under metabolic stress and therapy resistance.

Although direct validation in human DTCs is still limited, different functional studies demonstrated that pharmacological or genetic inhibition of ACSL3 in preclinical models restores PUFAs incorporation into membrane phospholipids, leading to an increase in lipid peroxidation, and enhancing sensitivity of dormant DTCs to ferroptosis [28,29]. These findings highlight the critical role of ACSL3 in supporting dormancy-associated therapy resistance and identify this enzyme as a potentially actionable metabolic target. Moreover, these data suggest that ACSL3 expression may serve as a potential biomarker to identify dormant tumor cells reservoirs that are susceptible to lipid metabolism-targeted strategies aimed at preventing metastatic relapses.

Notably, lipid metabolic rewiring can result in opposite ferroptosis outcome depending on BC subtypes. In particular, a subset of triple negative breast cancer (TNBCs)—in contrast to HR+ dormant DTCs—show elevated PUFAs biosynthesis which represents a specific vulnerability. Specifically, a study identified fatty acid desaturases 1 and 2 (FADS1/2)—which are key enzymes responsible for PUFAs biosynthesis—as highly expressed in a TNBC subset associated with a poorer prognosis [30]. Lipidomic analysis combined with functional metabolic assays revealed that FADS1/2 high-expressing TNBCs are intrinsically more susceptible to ferroptosis-inducing agents due to their abundance of PUFAs, which represent the substrates for iron-dependent lipid peroxidation [30]. Consequently, targeting FADS1/2, via both genetic interference and a pharmacological approach, makes these tumors ferroptosis-resistant, while unbalancing the PUFAs/MUFAs ratio through exogenous PUFAs supplementation re-sensitized resistant tumors to ferroptosis [30]. These findings highlight how lipid metabolism deregulation in TNBCs—in this case, increased PUFAs biosynthesis—can act as a context-dependent vulnerability biomarker.

Furthermore, an additional strategy to potentiate ferroptosis in these tumors was identified in the inhibition of lipid droplets (LDs) formation and turnover [31]. LDs function as a critical buffer, sequestering free fatty acids and peroxidized lipids [30,31]. Suppressing the buffering capacity of LDs was shown to enhance iron-dependent cell death in FADS1/2 high-expressing cells [31]. These findings, which have been validated in vitro, in vivo in clinically relevant mouse- and human-derived models, and also in a retrospective cohort of TNBC patients, suggest that FADS1/2 and LDs regulation are promising targets to overcome treatment resistance and improve prognosis in TNBC patients.

The possibility of targeting ferroptosis in TNBCs is further supported by a recent study, which highlights the potential of this peculiar pathway as a broad therapeutic strategy for this challenging subtype [32]. Comprehensive investigations into the metabolic and redox imbalances in TNBCs have identified several critical regulatory pathways, whose dysregulation contributes to cancer cell survival and resistance [32]. In particular, inducing ferroptosis—through inhibition of key antioxidant defenses including GPX4 inhibitors, or system Xc- inhibitors—has been demonstrated to inhibit tumor growth, overcome drug resistance, and enhance the efficacy of conventional therapies in TNBC models [32]. In contrast, lipophilic antioxidants act as ferroptosis inhibitors by preventing lipid peroxidation and are commonly used experimentally to block ferroptotic cell death.

Moreover, additional regulatory pathways have been shown to modulate ferroptosis sensitivity in TNBC. These include the Nuclear Factor Erythroid 2-related Factor 2 (NRF2) antioxidant response pathway, which transcriptionally upregulates multiple redox-protective genes, and ferroptosis suppressor protein 1 (FSP1). This protein acts limiting lipid peroxidation independently of GPX4 through CoQ10 regeneration. Hyperactivation of these antioxidant networks contributes to ferroptosis and tumor cell survival under oxidative stress conditions [32].

Accordingly, therapeutic strategies aimed at inducing ferroptosis in TNBCs rely on disabling antioxidant defenses rather than reinforcing them, also suggesting that targeting ferroptosis represents a highly promising strategy to improve treatment outcomes for TNBCs [32]. Moreover, while further research is essential to fully elucidate the underlying mechanisms and optimize safety and efficacy, these results also highlight the context-dependent role of lipid metabolism in ferroptosis regulation.

In addition to intrinsic metabolic adaptations, the tumor microenvironment (TME) also has an active role in shaping ferroptosis resistance in dormant DTCs. In adipocyte-rich metastatic niches, such as bone marrow, stromal adipocytes provide abundant exogenous fatty acids—particularly oleic acid—that are transferred to DTCs and incorporated into membrane phospholipids in an ACSL3-dependent way. This lipid transfer not only supports energy balance and membrane remodeling, but also limits lipid peroxidation and ferroptosis sensitivity. Interestingly, this metabolic crosstalk represents a potential therapeutic vulnerability, in fact, disrupting fatty acid availability, adipocyte–tumor cell lipid transfer, or ACSL3-mediated incorporation may weaken the protective niche and sensitize dormant DTCs to ferroptosis induction [19].

Considering all these new findings, future studies are needed to better understand the regulatory mechanisms controlling ACSL3 expression across breast cancer subtypes, as well as the potential interactions with other antioxidant pathways such as the GPX4–glutathione axis, and the dynamics of lipid metabolism in various metastatic niches. Integrative metabolomic and pharmacological approaches will also be useful to help uncover novel dormancy-specific metabolic targets and advancing therapies aimed at eradicating dormant tumor reservoirs.

## 3. SCD1-Driven MUFAs Synthesis as a Ferroptosis Resistance Mechanism in Breast Cancer Dormancy

Stearoyl-CoA desaturase-1 (SCD1) plays a central role in cellular lipid metabolism, being involved in the de novo desaturation of saturated fatty acids (SFAs) into monounsaturated fatty acids (MUFAs), specifically oleic and palmitoleic acids. Unlike ACSL3, which mediates the activation and incorporation of exogenous MUFAs derived from the microenvironment, SCD1 controls the endogenous generation of MUFAs. Moreover, membrane fluidity, lipid droplets biogenesis, and the cellular redox status are significantly influenced by this enzymatic activity, highlighting its role in ferroptosis. In breast cancer, SCD1 overexpression has been observed across the different subtypes: HER2-positive, triple-negative, and HR+ lesions, where its activity has been associated with tumor cell survival under metabolic and oxidative stress conditions [31,33]. In particular, HR+ breast cancer—which represents most breast tumors cases—is characterized by a high rate of late relapse related to the presence of dormant DTCs. Evidence from preclinical breast cancer models and related systems suggest that SCD1-dependent MUFAs synthesis supports long-term survival of dormant DTCs by protecting them from ferroptosis, and also promoting metastatic outgrowth. In fact, in hostile microenvironments, such as bone marrow and adipose-rich niches, endogenous MUFAs production via SCD1 is important to maintain membrane integrity, since they can reduce lipid peroxidation, enabling the survival of dormant HR+ cells in this context [34,35]. Furthermore, it is important to highlight that DTCs are characterized by high plasticity and selective lipid metabolic activity, which enable them to survive under oxidative stress, with these mechanisms being completely unrelated to cell proliferation.

Also, it has been demonstrated that estrogen-dependent signaling may influence lipid metabolism, potentially modulating both SCD1 expression and activity in HR+ tumors, with a further impairment of ferroptosis [36]. Specifically, estrogen receptor α (ERα) signaling has been implicated in the regulation of lipid metabolism via sterol regulatory element-binding protein 1 (SREBP1), a pivotal transcriptional regulator of lipogenic genes. In HR+ breast cancer models, the ERα–SREBP1 axis can promote SCD1 transcription, thus enhancing endogenous MUFAs synthesis and reinforcing ferroptosis resistance under metabolic and oxidative stress conditions [37].

**Figure 2:**
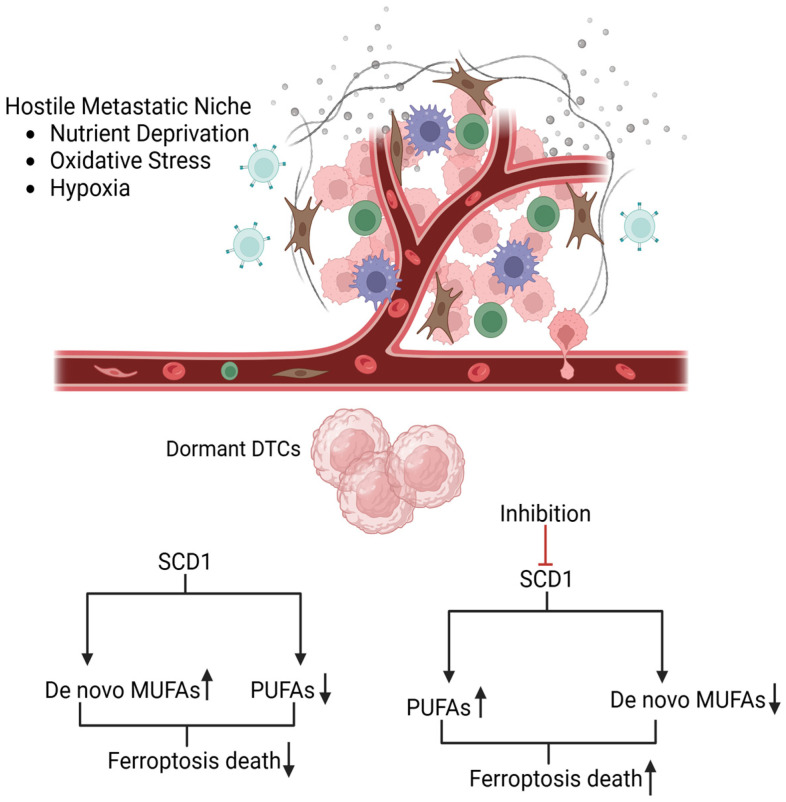
Lipid metabolic rewiring and ferroptosis resistance in dormant DTCs. Dormant disseminated tumor cells (DTCs) adapt to hostile metastatic niches by reshaping lipid metabolism through multiple coordinated mechanisms. Among these, stearoyl-CoA desaturase-1 (SCD1)-driven lipid desaturation promotes monounsaturated fatty acids (MUFAs) enrichment, with a reduction in polyunsaturated fatty acids (PUFAs) within cellular membranes, thus limiting ferroptosis sensitivity. Inhibition of SCD1 increases membrane PUFAs content and enhances ferroptosis sensitivity in dormant DTCs. Created in BioRender. Bertero, L. (2026) https://BioRender.com/gxhgido.

In preclinical models of breast cancer dormancy, SCD1-dependent MUFAs biosynthesis represents an important protective mechanism that prevents ferroptosis death in dormant DTCs. In fact, through the enzymatic desaturation of saturated fatty acids, SCD1 enriches cell membranes with MUFAs, while reducing the incorporation of PUFAs, that are highly susceptible to lipid peroxidation (Figure 2). This type of remodeling reduces the generation of lipid-derived reactive oxygen species (ROS), thus protecting the cells even under conditions of glutathione peroxidase 4 (GPX4) inhibition, that is known to be a canonical ferroptosis defense mechanism [34,38]. Moreover, this lipid rearrangement is involved in both preserving the membrane integrity and lipid droplets formation, which have the function of storing potentially toxic lipids, thus reducing the oxidative damage.

Interestingly, there are several studies in breast cancer models that show how pharmacological inhibition of SCD1 impairs the protective lipid membrane structure and restores ferroptosis sensitivity. In fact, targeting this enzyme promotes the restoration of PUFAs incorporation into the cell membrane, leading to an increase in lipid peroxidation and consequently enhancing ferroptosis sensitivity of cells. For example, in MDA-MB-468 cells the blockade of SCD1 using the inhibitor CAY10566 results in a reduction in MUFAs availability in the tumor microenvironment, reducing colony formation, and enhancing ferroptosis sensitivity [39]. Moreover, it was also observed that shRNA-mediated knockdown or pharmacological inhibition of SCD1 in MDA-MB-231 models impaired spheroid growth and sensitized cells to ROS-induced ferroptosis, highlighting the enzyme’s critical role in sustaining redox balance and survival [34]. Other literature data on preclinical models show that blocking SCD1 impairs metastatic outgrowth, highlighting its therapeutic potential in targeting minimal residual disease and preventing metastatic relapses [40].

Together, these findings support SCD1 role as a metabolic marker of dormancy-associated ferroptosis resistance and as a therapeutic target, which is of particular interest in aggressive breast cancer where dormancy and therapy resistance still represent an unmet clinical need. It is important to note that this pathway complements the ACSL3-mediated incorporation of exogenous MUFAs, suggesting that dormant DTCs exploit both intracellular synthesis and microenvironment-derived lipid resources to maintain a MUFAs-rich, ferroptosis-resistant state.

Ongoing research aims to elucidate the regulatory networks governing SCD1 expression and activity during dormancy, including hypoxia-inducible factors and lipid-sensing nuclear receptors. Understanding these pathways could support the development of combination therapies that promote ferroptosis sensitivity by co-targeting SCD1 and key antioxidant systems.

## 4. Lipid Droplets as Redox-Buffering Organelles in Dormant Breast Cancer Cells

**Figure 3:**
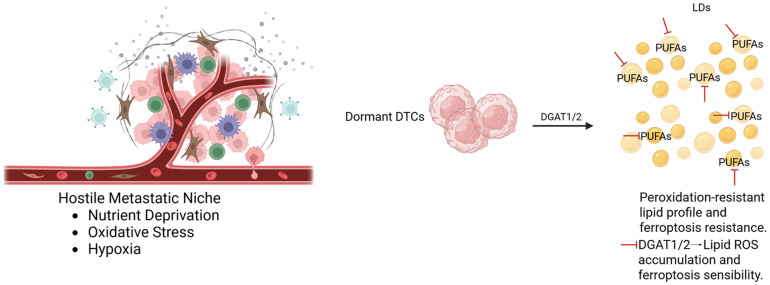
Lipid droplets biosynthesis and ferroptosis resistance in dormant DTCs. Accumulation of lipid droplets (LDs), promoted by DGAT1/2-dependent triglyceride synthesis, provides protection against ferroptosis by sequestering oxidable lipids and limiting lipid peroxidation. This mechanism supports long-term survival, therapy resistance, and late metastatic relapse. Inhibition of DGAT1/2 impairs LDs formation, leading to lipid-derived reactive oxygen species (ROS) accumulation and induction of ferroptosis in dormant DTCs. Created in BioRender. Bertero, L. (2026) https://BioRender.com/qxml3lz.

Lipid droplets (LDs) are active intracellular organelles that can accumulate neutral lipids, playing a key role in regulating cellular redox homeostasis. LDs can also store PUFAs within triacylglycerol (TAG) cores, reducing the pool of oxidizable lipids available for membrane biosynthesis and therefore protecting the cancer cells from ferroptosis [40]. In addition, these droplets can also accumulate MUFAs synthesized by SCD1 and activated by ACSL3, helping to maintain a peroxidation-resistant lipid profile [14]. In fact, in models of dormant DTCs, metabolic adaptations including increased lipid synthesis and storage are significantly enhanced. Dormant DTCs upregulate de novo lipogenesis and exhibit profound changes in lipid handling which support survival under metabolic and oxidative stress, as well as being consistent with enhanced LDs accumulation as a protective adaptation (Figure 3). Experimental evidence shows that lipid droplet biogenesis limits oxidative damage and sequesters toxic lipid species, thereby promoting DTCs survival in hostile conditions [41,42].

Moreover, it has been demonstrated that in acidic microenvironmental conditions, which is common in many metastatic niches, LDs biogenesis is upregulated via DGAT1/2 mediated pathways. These mechanisms promote TAG formation and PUFAs storage, resulting in protection against ferroptosis damage in dormant DTCs [42]. So, pharmacological blockade of DGAT activity is associated with the impairment of this protective mechanism. Specifically, PUFAs are reincorporated into the cell membranes, leading to lipid-derived ROS accumulation and ferroptosis cell death. Interestingly, this process is reversible by ferroptosis inhibitors like ferrostatin-1 and liproxstatin-1, highlighting the protective role of LDs [43].

Among breast cancer subtypes, in HR+ lesions, dormant DTCs show a strong dependence on lipid-based adaptations, including lipid storage and antioxidant defenses, to survive in challenging niche microenvironments such as bone marrow [44,45,46]. There is evidence that LDs play a protective role in these cells through the storage of MUFAs, thus preventing peroxidative damage and ferroptosis death. Furthermore, ER signaling has been shown to modulate enzymes with a pivotal role in lipid metabolism, including SCD1 and DGAT, thereby connecting hormonal signaling to ferroptosis resistance through LDs [47].

In vivo studies on breast cancer models further demonstrate that DGAT inhibition, particularly when combined with dietary PUFAs enrichment, can suppress tumor growth in xenograft models via ferroptosis-mediated mechanisms. An example is in TNBCs, where adipocyte-derived oleic acid promotes LDs formation and ferroptosis resistance through ACSL3 activity, highlighting how stromal interactions are involved in reinforcing metabolic plasticity in dormant cancer cells [48,49].

Together, all these findings define lipid droplets as active regulators of ferroptosis. For this reason, therapeutic strategies targeting LDs biogenesis in combination with ferroptosis inducers may represent a promising way to eliminate dormant DTCs and prevent metastatic relapses, particularly in HR+ breast cancer where endocrine and metabolic signaling are intertwined.

## 5. The GPX4–Glutathione Axis in the Regulation of Lipid Peroxidation in Dormant Breast Cancer Cells

The glutathione peroxidase 4 (GPX4)–glutathione (GSH) axis plays a pivotal role in antioxidant pathways and is, consequently, involved in preventing ferroptosis. Specifically, GPX4 is a selenoprotein that enzymatically reduces phospholipid hydroperoxides into non-toxic lipid alcohols using GSH as a cofactor and effectively reducing lipid peroxidation [28,50]. Dormant DTCs are characterized by high levels of GPX4 and GSH, and this feature enables them to withstand chronic oxidative and metabolic stress, which is associated with the dormant, minimal-residual state [28].

Experimental inhibition of GPX4 activity using for example RSL-3 or blocking of cystine import through system Xc- (e.g., erastin targeting SLC7A11) triggers lethal lipid-derived ROS accumulation and ferroptotic cell death [51,52]. When these approaches are combined with pharmacological impairment of lipid-based defense mechanisms, such as MUFAs enrichment or LDs biogenesis, the ferroptotic response is further amplified, highlighting the critical role of GPX4-GSH axis in dormancy-associated ferroptosis resistance [52]. Focusing on breast cancer cells, they often overexpress the SLC7A11-functional subunit of the cysteine/glutamate antiporter system Xc- and GPX4 in order to survive in stressful environmental conditions. Accordingly, different studies demonstrate that the higher expression of these two markers is associated with an increase in sensitivity to ferroptosis inducers, such as erastin and RLS-3 [52,53,54,55]. Interestingly, this metabolic dependency is specifically evident in HR+ breast cancer. In fact, it was observed that in luminal (ER+, PR+) cell lines like MCF-7 and T-47D, treatment with abemaciclib—a CDK4/6 inhibitor—leads to a compensatory upregulation of GPX4 expression, resulting in a higher protection against therapy-induced oxidative stress [56,57]. However, this adaptive response also reveals a therapeutic vulnerability: when silencing GPX4 through CRISPR-CAS9 technique or combining abemaciclib with RSL-3, a synergistical induction of ferroptosis can be observed in these cells, offering a potential strategy to target HR+ dormant DTCs [23,24].

Furthermore, GPX4 proper maturation and functional activity depends on selenocysteine insertion via the Sec-tRNA^sec^ synthesis pathway, with enzymes such as SEPHS2, SEPSECS, and PSTK which have a key role in this pathway. There is evidence showing how the knockdown of these enzymes in TNBC cells significantly lowers GPX4 levels, inducing ferroptosis, and impairing metastatic colonization in vivo, thus highlighting their pivotal role in GPX4 antioxidant activity [58,59].

All these results show that GPX4-GSH axis works in close synergy with lipid-based defense mechanisms, such as the enrichment in MUFAs content in cells membrane and LDs sequestration, creating a solid, multi-layered system to protect dormant DTCs from ferroptosis [51,52]. Particularly in HR+ breast cancer, where endocrine signaling is metabolically intertwined with redox regulation, the up-regulation of GPX4 as an answer to hormonal or cell-cycle-targeted therapies reflects not only an adaptive response but also a vulnerability that may be exploited through ferroptosis-based therapeutic approaches [59,60].

**Figure 4:**
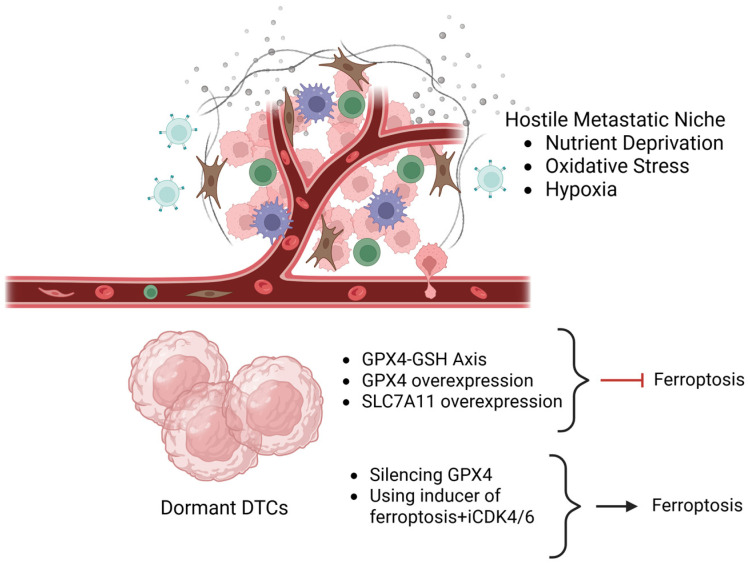
Antioxidant axis role in regulating ferroptosis resistance in dormant DTCs. Reinforced antioxidant defenses including the glutathione peroxidase 4 (GPX4)–glutathione (GSH) axis and cysteine/glutamate antiporter SLC7A11, limit lipid peroxidation and suppress ferroptosis in dormant disseminated tumor cells (DTCs). These defenses contribute to long-term cellular survival, therapy resistance and late metastatic relapse. Pharmacological inhibition of antioxidant pathways disrupts redox homeostasis and promotes ferroptosis. Created in BioRender. Bertero, L. (2026) https://BioRender.com/ck0oj1i.

Targeting this axis through different strategies, such as GPX4 inhibition, cystine import blockade, or impairment of Sec-tRNA^sec^ pathways, offers a powerful therapeutic option. Strategic combinations that also impair lipid metabolic defenses, as for example MUFAs synthesis, SCD1/ACSL3 activity and LDs biogenesis, are promising for selectively targeting dormant DTCs, and promoting ferroptosis death in these cells, thus preventing metastatic relapses in breast cancer (Figure 4).

### Additional Ferroptosis-Regulatory Pathways in Dormant DTCs

In parallel with the GPX4–glutathione axis, several alternative mechanisms regulate ferroptosis sensitivity. For example, the FSP1–CoQ10 pathway provides a GPX4-independent defense by regenerating reduced coenzyme Q10 at the plasma membrane, limiting lipid peroxidation [57,60]. Similarly, dihydroorotate dehydrogenase (DHODH) acts as a mitochondrial ferroptosis suppressor by supporting ubiquinol regeneration in mitochondrial membranes, thereby protecting against oxidative damage even when GPX4 activity is compromised [61].

Phospholipid remodeling enzymes such as LPCAT3, together with phospholipases including iPLA2β, modulate the abundance of oxidizable polyunsaturated fatty acids in membrane phospholipids, influencing ferroptosis sensitivity through control of PUFAs-rich phospholipid pools [62]. Although the exact roles of these pathways in dormant DTCs remain to be fully elucidated, emerging evidence suggests that they may cooperate with lipid metabolic rewiring and antioxidant systems to support cell survival under oxidative stress.

Integrating these mechanisms enhances the completeness of the proposed model of ferroptosis regulation in dormant DTCs and highlights potential metabolic vulnerabilities that could be therapeutically targeted (Table 1).

## 6. Conclusions and Future Directions

The persistence of dormant disseminated tumor cells (DTCs) represents a major clinical challenge in breast cancer, particularly in hormone receptor-positive (HR+) subtypes, where the risk of late recurrence still remains high. Increasing evidence shows that dormancy is not a passive state of metabolic quiescence, but rather an active condition supported by highly coordinated metabolic and redox adaptations. In this context, lipid metabolism has emerged as a critical survival strategy for these cells, enabling adaptation to hostile microenvironments through different mechanisms, including the enrichment of membrane monounsaturated fatty acids (MUFAs), lipid droplets (LDs) biogenesis, and antioxidant defense activation, such as the GPX4–glutathione axis. These mechanisms cooperatively suppress lipid peroxidation and ferroptosis, supporting long-term cellular quiescence and resistance to conventional therapies.

Interestingly, the metabolic plasticity that sustains dormancy and promotes metastatic relapses in breast cancer also unveils actionable vulnerabilities. In fact, preclinical studies demonstrate that pharmacological impairment of lipid metabolic defenses—through inhibition of key enzymes such as ACSL3 and SCD1, lipid droplets biogenesis and GPX4 activity—can overcome these mechanisms and selectively induce death in dormant breast cancer cells. These effects may be further enhanced by combining ferroptosis inducers or redox-disruptors agents. For example, in HR+ disease, where endocrine therapies and CDK4/6 inhibitors can reshape both redox and lipid metabolism by imposing chronic stress, ferroptosis-based strategies may be particularly effective in targeting minimal residual disease, which still represents an unmet clinical need. In this context, targeting metabolic crosstalk within the tumor microenvironment—particularly fatty acid supply from bone marrow adipocytes—may further enhance ferroptosis sensitivity of dormant DTCs.

In addition to mechanistic insights, the diagnostic potential of ferroptosis-related metabolic states is an expanding area of interest. In fact, the emerging role of lipid metabolic rewiring and ferroptosis resistance in dormant DTCs also raises important diagnostic and translational opportunities. Advances in lipidomic profiling may enable the characterization of membrane lipid composition and oxidizable lipid species in DTCs, potentially revealing signatures associated with ferroptosis resistance [63]. In parallel, key regulators of ferroptosis such as GPX4 and SLC7A11 have been detected in human cancer tissues by immunohistochemistry (IHC) and correlated with clinical outcomes, suggesting their potential as tissue-based biomarkers in diagnostic contexts [64]. Moreover, biochemical indicators related to ferroptosis—including lipid peroxidation products (e.g., malondialdehyde, 4-HNE) and redox balance markers such as glutathione levels—can be measured in patient serum or tissue samples and may complement molecular profiling in stratifying metabolic states [65].

In addition to tissue analysis, liquid biopsy approaches—including circulating tumor DNA (ctDNA), circulating tumor cells (CTCs), and potentially DTCs-derived material—may provide minimally invasive tools to monitor lipid metabolic rewiring or ferroptosis sensitivity over time, aiding patient stratification and treatment response assessment. These biomarkers could also be correlated with clinically relevant endpoints, including late metastatic recurrence, duration of dormancy/minimal residual disease, and response to endocrine or targeted therapies.

From a translational perspective, however, several challenges must be addressed before these strategies can be implemented clinically. Systemic inhibition of broadly expressed regulators such as GPX4 or SCD1, may lead to toxicity in normal tissues that rely on lipid metabolism and redox homeostasis, thus limiting the therapeutic landscape. Consequently, future approaches will need to prioritize tumor-selective or context-dependent strategies. These may include exploiting dormancy-specific metabolic states, optimizing treatment timing or integrating ferroptosis-inducing agents with existing endocrine therapies to achieve selective vulnerability of dormant DTCs, while minimizing systemic toxicity.

To date, evidence supporting these targets derives from a combination of in vitro studies, in vivo preclinical models and retrospective analyses of human tumor cohorts, highlighting both their biological relevance and the need for prospective clinical validation. Looking ahead, several key questions need further investigation. These include elucidating the molecular regulation of lipid metabolism during dormancy across breast cancer subtypes and defining the dynamic interplay between tumor cells and metastatic niche in shaping ferroptosis sensitivity. Future studies leveraging patient-derived DTCs, single-cell lipidomic, and spatial profiling approaches will be essential to validate these mechanisms in human dormancy and to uncover dormancy-specific metabolic signatures.

Ultimately, translating these insights into clinical applications will surely require rigorous in vivo validation and well-designed early-phase trials for the development of safe and effective ferroptosis-inducing agents, that can be used in combination with existing endocrine and target therapies to reduce the risk of metastatic relapse. Moreover, targeting lipid metabolism-driven ferroptosis resistance can offer a compelling opportunity to redefine the management of breast cancer dormancy, particularly in HR+ breast lesions.

Shifting therapeutic approaches toward the elimination of residual disease before metastatic outgrowth holds potential not only to reduce late relapses but also to significantly improve long-term survival and quality of life for patients with breast cancer.

### Integrated Model of Lipid Metabolic Rewiring and Ferroptosis Resistance in Dormant DTCs

Dormant DTCs adapt to hostile metastatic niches through coordinated modulation of lipid uptake, synthesis, storage, and antioxidant defenses, collectively shaping a ferroptosis-resistant state.

Specifically, ACSL3-mediated activation and incorporation of exogenous MUFAs supplied by the microenvironment, cooperates with SCD1-driven de novo MUFAs synthesis to promote a membrane lipid composition enriched in monounsaturated fatty acids and depleted in peroxidation-prone PUFAs. In parallel, lipid droplet biogenesis, supported by DGAT1/2 activity, buffers excess fatty acids and limits lipid peroxidation, while antioxidant systems such as the GPX4–glutathione axis and SLC7A11-mediated cystine uptake suppress lethal lipid ROS accumulation.

This integrated framework also highlights multiple actionable vulnerabilities, including ACSL3 and SCD1 inhibitors, DGAT1/2 inhibitors, and ferroptosis inducers targeting system Xc- (e.g., erastin) or GPX4 (e.g., RSL-3) which can restore sensitivity to this mechanism in dormant DTCs.

**Figure 5:**
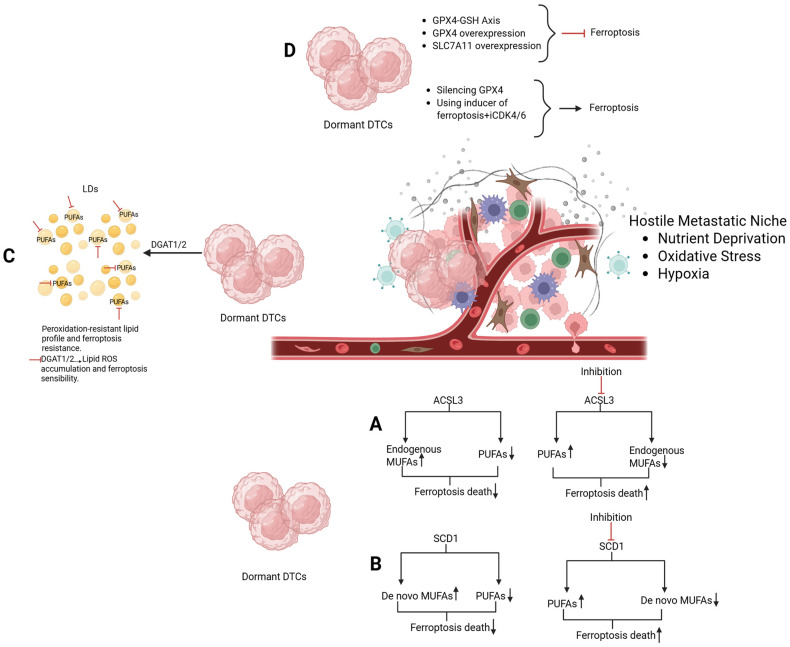
Integrated model of lipid metabolic rewiring and ferroptosis resistance in dormant disseminated tumor cells (DTCs). (**A**) Acyl-CoA synthetase long-chain family member 3 (ACSL3) mediates the activation and incorporation of exogenous monounsaturated fatty acids (MUFAs) supplied by the tumor microenvironment into membrane phospholipids, reducing the availability of peroxidation-prone polyunsaturated fatty acids (PUFAs). (**B**) Stearoyl-CoA desaturase 1 (SCD1) promotes de novo endogenous MUFAs synthesis from saturated fatty acids, further enriching membrane MUFAs and limiting lipid peroxidation. (**C**) Lipid droplets (LDs) biogenesis, supported by DGAT1/2 activity, buffers excess fatty acids and sequesters oxidizable lipids, contributing to redox homeostasis and long-term cellular quiescence. (**D**) Reinforced antioxidant systems, including the GPX4–glutathione axis and SLC7A11-mediated cystine uptake, suppress lipid peroxidation and ferroptosis. Together, these interconnected pathways cooperatively sustain dormancy, therapy resistance, and late metastatic relapse, while highlighting multiple actionable vulnerabilities for ferroptosis-based therapeutic strategies. Created in BioRender. Bertero, L. (2026) https://BioRender.com/4hhvzcf.

The schematic model in Figure 5 provides a visual platform to identify potential therapeutic targets and rational combination strategies aimed at eliminating minimal residual disease and preventing late metastatic relapses in breast cancer.

## Figures and Tables

**Table 1:** Therapeutic targeting of lipid metabolism and ferroptosis resistance in dormant breast cancer cells.

Target/Pathway	Proposed Intervention Class	Representative Agents	Evidence Level	Expected Toxicities/Limitations	Candidate Biomarkers for Response
ACSL3-mediated MUFAs activation and incorporation	Inhibition of fatty acid activation and membrane lipid remodeling	Triacsin C (preclinical)	In vitro and in vivo preclinical dormancy models	Potential metabolic toxicity due to ACSL3 expression in normal lipid-metabolizing tissues; lack of clinically approved inhibitors	ACSL3 expression; membrane MUFAs/PUFAs ratio; lipid peroxidation markers
SCD1-driven de novo MUFAs synthesis	Enzymatic inhibition of fatty acid desaturation	CAY10566, A939572 (preclinical)	In vitro and in vivo breast cancer models	Systemic metabolic toxicity; impact on physiological lipid homeostasis	SCD1 expression; lipid desaturation index; MUFAs enrichment
Lipid droplet (LD) biogenesis via DGAT1/2	Inhibition of neutral lipid storage	DGAT1/2 inhibitors (preclinical)	In vitro and in vivo cancer models	Disruption of lipid buffering in normal cells; compensatory lipid pathways	LD abundance; DGAT1/2 expression; intracellular free fatty acid levels
GPX4–glutathione antioxidant axis	Ferroptosis induction via GPX4 inhibition	RSL-3	In vitro and in vivo	Narrow therapeutic window; high systemic toxicity due to GPX4 essentiality	GPX4 expression; lipid peroxidation products (MDA, 4-HNE)
System Xc-(SLC7A11-mediated cystine uptake)	Inhibition of cystine import and glutathione synthesis	Erastin	In vitro and in vivo; limited clinical exploration	Redox imbalance in normal tissues; off-target effects	SLC7A11 expression; glutathione levels; oxidative stress markers
Combination strategies (lipid metabolism + redox stress)	Combined targeting of lipid defenses and ferroptosis pathways	CDK4/6 inhibitors + GPX4 or System Xc- inhibitors (preclinical)	Preclinical	Optimization of dosing and scheduling required; cumulative toxicity	Integrated lipid–redox metabolic signatures; therapy-induced stress markers

## Data Availability

No new data were created or analyzed in this study. Data sharing is not applicable to this article.
